# Discriminative performance of pancreatic stone protein in predicting ICU mortality and infection severity in adult patients with infection: a systematic review and individual patient level meta-analysis

**DOI:** 10.1007/s15010-023-02093-w

**Published:** 2023-09-14

**Authors:** Patrick Zuercher, André Moser, Luis Garcia de Guadiana-Romualdo, Martin J. Llewelyn, Rolf Graf, Theresia Reding, Philippe Eggimann, Yok-Ai Que, Josef Prazak

**Affiliations:** 1 Department of Intensive Care Medicine, INO E-104, Inselspital, Bern University Hospital, University of Bern, CH-3010 Bern, Switzerland; 2 CTU Bern, University of Bern, Bern, Switzerland; 3 Laboratory Medicine Department, Santa Lucia University Hospital, Cartagena, Spain; 4 University Hospitals Sussex NHS Foundation Trust, Brighton BN2 5BE UK and Brighton and Sussex Medical School, Falmer, BN1 9PS UK; 5 Department of Visceral and Transplantation Surgery, Universitätsspital Zürich, Zurich, Switzerland; 6 Department of Locomotor Apparatus, Lausanne University Hospital (CHUV) and University of Lausanne, Lausanne, Switzerland

**Keywords:** Pancreatic stone protein, PSP, Infection, Mortality, Biomarker

## Abstract

**Background:**

Several studies suggested pancreatic stone protein (PSP) as a promising biomarker to predict mortality among patients with severe infection. The objective of the study was to evaluate the performance of PSP in predicting intensive care unit (ICU) mortality and infection severity among critically ill adults admitted to the hospital for infection.

**Methods:**

A systematic search across Cochrane Central Register of Controlled Trials and MEDLINE databases (1966 to February 2022) for studies on PSP published in English using ‘pancreatic stone protein’, ‘PSP’, ‘regenerative protein’, ‘lithostatin’ combined with ‘infection’ and ‘sepsis’ found 46 records. The search was restricted to the five trials that measured PSP using the enzyme-linked immunosorbent assay technique (ELISA). We used Bayesian hierarchical regression models for pooled estimates and to predict mortality or disease severity using PSP, C-Reactive Protein (CRP) and procalcitonin (PCT) as main predictor. We used statistical discriminative measures, such as the area under the receiver operating characteristic curve (AUC) and classification plots.

**Results:**

Among the 678 patients included, the pooled ICU mortality was 17.8% (95% prediction interval 4.1% to 54.6%) with a between-study heterogeneity (I-squared 87%). PSP was strongly associated with ICU mortality (OR = 2.7, 95% credible interval (CrI) [1.3–6.0] per one standard deviation increase; age, gender and sepsis severity adjusted OR = 1.5, 95% CrI [0.98–2.8]). The AUC was 0.69 for PSP 95% confidence interval (CI) [0.64–0.74], 0.61 [0.56–0.66] for PCT and 0.52 [0.47–0.57] for CRP. The sensitivity was 0.96, 0.52, 0.30 for risk thresholds 0.1, 0.2 and 0.3; respective false positive rate values were 0.84, 0.25, 0.10.

**Conclusions:**

We found that PSP showed a very good discriminative ability for both investigated study endpoints ICU mortality and infection severity; better in comparison to CRP, similar to PCT. Combinations of biomarkers did not improve their predictive ability.

**Supplementary Information:**

The online version contains supplementary material available at 10.1007/s15010-023-02093-w.

## Background

The early recognition of patients with severe infections and potentially unfavorable outcome is critical to improve mortality in sepsis, as patients at high-risk of death might benefit from individualized care and advanced support [1]. Biomarkers are increasingly being used to target personalized care and precision medicine in various clinical settings [2–5], including for the management of sepsis [6, 7]. C-reactive protein (CRP) and procalcitonin (PCT) are broadly used to stratify infection according to disease severity and potential outcome despite their poor performance for that purpose [8–13]. Other biomarkers have been proposed, but their place in clinical practice is not established [14–16].

Pancreatic stone protein (PSP) has recently emerged as a promising biomarker of infection [17].

PSP is a globular polypeptide adopting a fold described for C-type lectins with a diverse range of functions, including signalling receptors in homeostasis and innate immunity, playing a crucial role in inflammatory response and leukocyte and platelet trafficking. It is mostly synthesized by the pancreas and the intestine with increasing blood levels early in the context of sepsis [17]. The point-of-care (POC) machines for bedside analysis only need a drop of whole blood to deliver results within few minutes [17].

Over the last two decades, PSP has been thoroughly evaluated in various medico-surgical patient populations and multiple clinical settings, especially in emergency rooms (ER), burn and intensive care units (ICUs) [18–23]. Several studies, including a recent meta-analysis [24], conducted in adults, children and neonates investigated the capacity of PSP to diagnose infection [20–22], characterize disease severity [19, 23] and predict outcome of patients with sepsis [19, 23, 25–27].

Here, we perform an individual patient level meta-analysis to evaluate the ability of PSP to predict patients with poor outcome and/or severe disease and report classification plots with continuous risk thresholds to support clinical decision-making based on current recommendations for predictions models [34].

## Methods

### Search strategy and selection criteria

A systematic literature search was performed across the Cochrane Central Register of Controlled Trials (CENTRAL and MEDLINE (1966 to February 2022) databases using “pancreatic stone protein”, “PSP”, “regenerative protein”, “infection”, “sepsis”, “lithostatin” as keywords and/or MeSH Terms. The search strategy was prepared according to PRISMA individual patient data guidelines (Supplemental Tables 1 and 2) [28]. The search was restricted to original human clinical trials on PSP/reg published in English before February 2022 that evaluated the performance of PSP for the assessment of the severity of infection as well as for predicting its outcome among unselected adult patients upon their admission to the ED or the ICU. The search was further restricted to studies that determined PSP levels in blood using the enzyme-linked immunosorbent assay technique (ELISA) developed and described by Rolf Graf et al*.* [20, 29], to impede calculation limitations when plotting equal PSP levels when using different analysing methods. Paediatric trials and autopsy studies were excluded. The definitions of infection used in each of the eligible studies are presented in Supplemental Table 3.

**Fig. 1:**
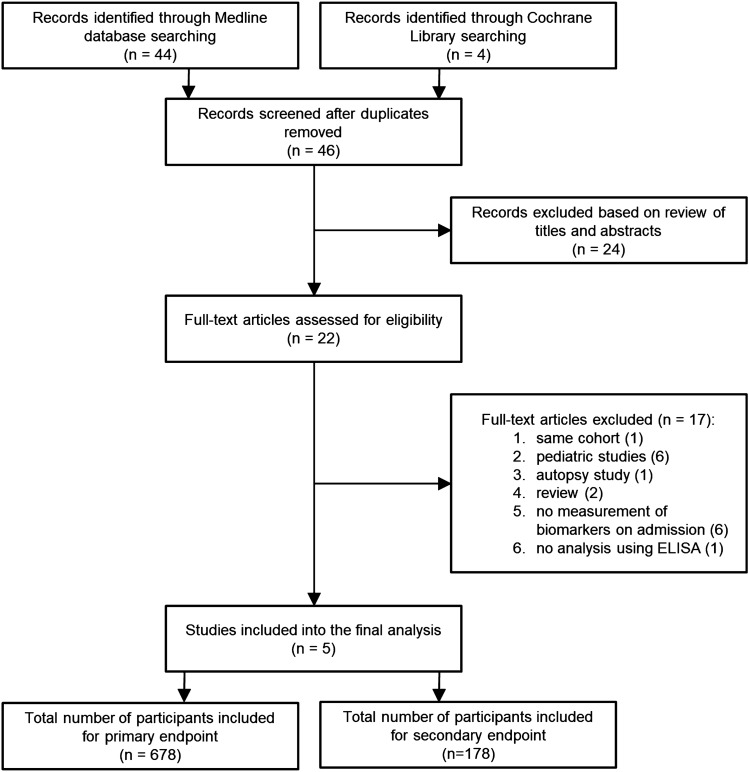
Study flowchart

Two reviewers (JP and YAQ) independently assessed trial eligibility based on titles, abstracts, full-text reports, and further information from investigators as needed (Fig. 1). Study protocols and unedited databases containing anonymized individual patient data were obtained from investigators of all eligible trials.

The study was registered on Prospero (#CRD42022308207). The Cantonal Ethical Committee of the State of Bern (#2018-01356_V2.1_25.2.2022) reviewed and approved the meta-analysis research protocol while the respective ethical committees already approved all individual studies.

### Assessment of data validity

All raw data were received from their principal investigators with patient specific anonymized ID and contained at least the following information: age, gender, Sequential Organ Failure Assessment (SOFA) Score and blood levels of PSP, CRP and PCT upon admission, days to death and ICU mortality. Data from each eligible study were first checked for duplicates and second against reported results. Queries were resolved with the principal investigator, trial data manager, or statistician whenever indicated.

### Study objectives

The primary objective of the study was to evaluate the diagnostic accuracy of PSP in predicting ICU mortality and compare it to CRP and PCT. The secondary objectives were: (i) to evaluate PSP ability to predict disease severity and compare it to CRP and PCT, and (ii) to explore whether different combinations of the three biomarkers further improve the prediction of ICU-mortality and disease severity.

### Study outcomes

Our primary endpoint was ICU mortality. Secondary outcomes were based on disease severity risk stratification on SOFA score upon admission: (i) non-complicated infection (patients with SOFA score ≤ 1; (ii) sepsis (patients with SOFA score ≥ 2) and (iii) septic shock (patients with SOFA score ≥ 2 and need for vasoactive drugs). We used the combined endpoint (sepsis and septic shock) as secondary outcome.

### Confounders

We adjusted all outcomes for age and sex. For the primary outcome ICU-mortality, we additionally adjusted for sepsis severity (mild moderate infection/infection, sepsis, septic shock).

### Statistical analysis

We described the study population by counts and percentages, median and interquartile range. Missing PSP, CRP or PCT measurements were replaced by median values within each study, because of the low missing value proportion: Percentage of missing values per study ranged from 0.4% for CRP/PCT to 5.6% for PSP (Supplemental Table 4). For adjusted analyses, three missing age values were replaced by the median value of the corresponding study.

We followed the meta-analytic approach used by *Prazak *et al*.* [24] and described in Steyerberg et al*.* [30]. Briefly, we evaluated three different models: (i) a random effect-random slope (RERS) model (random intercept on study and biomarkers as random slopes including a fixed effect on biomarkers for population mean interpretation of the random intercept and slope [31]); (ii) a random effect (RE) model (random intercept on study and fixed biomarker effect); and (iii) a fixed effect (FE) model (fixed biomarker effects without any patient clustering information). We compared models using the Akaike information criterion (AIC) and log-likelihoods. Because of the small number of studies and convergence issues of frequentist random effects models, we used Bayesian hierarchical logistic regression models. We used centered Gaussian priors with a standard deviation of 2.5 for intercept and biomarker effects [32]. For the centered multivariate Gaussian distributed random effects we used a Lewandowski-Kurowicka-Joe prior with a regularization parameter set to 1, a concentration parameter set to 1 and a unit-exponential prior on the scale parameters for the decomposition of the correlation matrix [2]. We used unadjusted models (using only biomarker values as predictors) and adjusted models (biomarker values and all confounding variables) reporting odds ratio with 95% credible intervals (CrI). Biomarker measurements were standardized (centered and divided by population standard deviation) and age centered and expressed as a 10-year increase. We reported study-specific outcome estimates and 95% CrI as well as between-study standard deviation and I-squared. 95% prediction intervals (PI) were calculated from the overall intercept plus a centered Gaussian distributed random variable with a standard deviation equal to the estimated between-study standard deviation. We reported AUC values with 95% confidence intervals (CIs), positive and negative predictive values, and classification plots [33]. A specific risk threshold cutoff was computed based on Youden’s index [34]. All analyses were performed in R version 4.1.2 [27]. Bayesian analyses were implemented in the Stan R interface [2] using 4 Markov chains with 1,000 warmup iterations per chain and 2,000 total iterations per chain.

## Results

### Study selection

Among the 48 records published before February 2022 and identified through the literature search, 46 full texts were further assessed for eligibility. 24 records were excluded based on review of title and abstracts. Mainly due to lack of measured biomarkers on admission or addressing a pediatric patient population, only five of the remaining 22 observational studies were included into the final analysis (Fig. 1 ; Table 1). Individual patient data from all patients were used for the evaluation of the primary endpoint ‘ICU mortality’ (Table 2). For the assessment of the secondary endpoint predicting disease severity, the studies of *Que *et al. [23] and *Guadiana-Romualdo *et al*.* (2019) [35] were excluded, since those studies only included patients with severe sepsis or septic shock (Fig. 1; Table 1).

**Table 1:** Characteristics of included studies

Study	Data collection period	Country	n	Eligibility	ICU mortality
Llewelyn et al. (2013)	Aug 2010 Jan 2011	UK	87	ICU or IMC patients	12 (14%)
Gukasjan et al. (2013)	Aug 2007 Feb 2010	CH	91	ICU patients with secondary peritonitis	23 (25%)
Que et al. (2015)	Sept 2009 May 2012	CH	249	Patients admitted to ICU for severe sepsis or septic shock due to various sources	81 (33%)
Guadiana-Romualdo et al. (2017)	Oct 2013 Nov 2013	E	129	ER patients	6 (4.7%)
Guadiana-Romualdo et al. (2019)	May 2013 May 2014	E	122	ICU patients	27 (22%)

*CH* Switzerland, *UK* United Kingdom, *E* Spain, *ICU *ntensive care unit, *IMC* Intermediate Care, *ER* Emergency Room

**Table 2:** Patient characteristics, by study

Characteristic*	Guadiana-Romualdo 2017 (*N* = 129)	Guadiana-Romualdo 2019 (*N* = 122)	Gukasjan 2013 (*N* = 91)	Llewelyn 2013 (*N* = 87)	Que 2015 (*N* = 249)	Overall (*N* = 678)
Age	67 (47, 79)	65 (53, 75)	66 (50, 72)	66 (54, 75)	63 (50, 76)	65 (51, 76)
Women	53 (41%)	54 (44%)	38 (42%)	0 (0%)	102 (41%)	247 (36%)
*Sepsis-3 classification*						
Non-complicated infection	82 (64%)	0 (0%)	30 (33%)	5 (5.7%)	0 (0%)	117 (17%)
Sepsis	37 (29%)	64 (52%)	35 (38%)	52 (60%)	93 (37%)	281 (41%)
Septic shock	10 (7.8%)	58 (48%)	26 (29%)	30 (34%)	156 (63%)	280 (41%)
ICU mortality	6 (4.7%)	27 (22%)	23 (25%)	12 (14%)	81 (33%)	149 (22%)
PSP	73 (33, 203)	436 (218, 620)	125 (26, 401)	116 (53, 250)	207 (62, 429)	185 (54, 410)
CRP	130 (67, 210)	210 (140, 318)	223 (144, 287)	146 (106, 203)	240 (139, 320)	190 (120, 290)
PCT	1 (0, 2)	13 (5, 35)	1 (0, 6)	3 (1, 9)	14 (3, 43)	5 (1, 24)

*Reported values represents either median (interquartile range) or *n* (%)

### Analysis population

We considered 678 patients in the study; 64% were male with a median age of 65 (Table 2). The biomarkers were measured on 549 patients admitted to ICUs and on 129 admitted to the emergency room. The distributions of the three biomarkers by study disease severity are shown in supplement (Supplemental Figs 1 and 2).

### ICU mortality

**Fig. 2:**
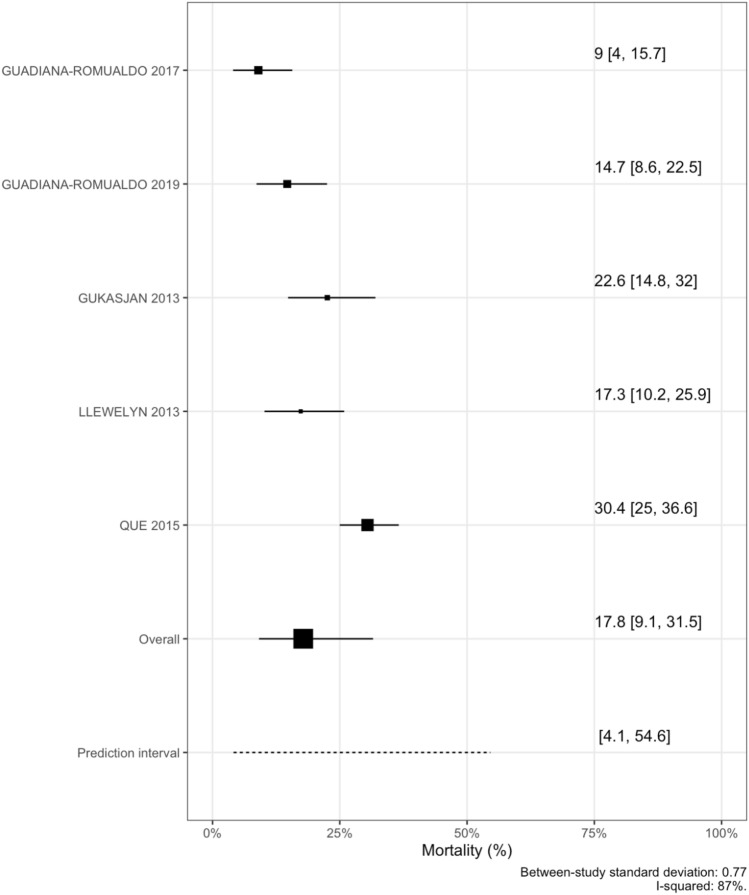
ICU mortality meta-analysis

The observed crude overall ICU mortality was 22% (149 out of 678 included patients). Model performance was best for a RERS models based on AIC (Supplemental Table 5). The pooled overall estimate from a RERS model was 17.8%, 95% CrI (9.1–31.5%) with a 95% PI ranging from 4.1–54.6% with a substantial heterogeneity between studies (*I-squared* 87%), (Fig. 2).

**Fig. 3:**
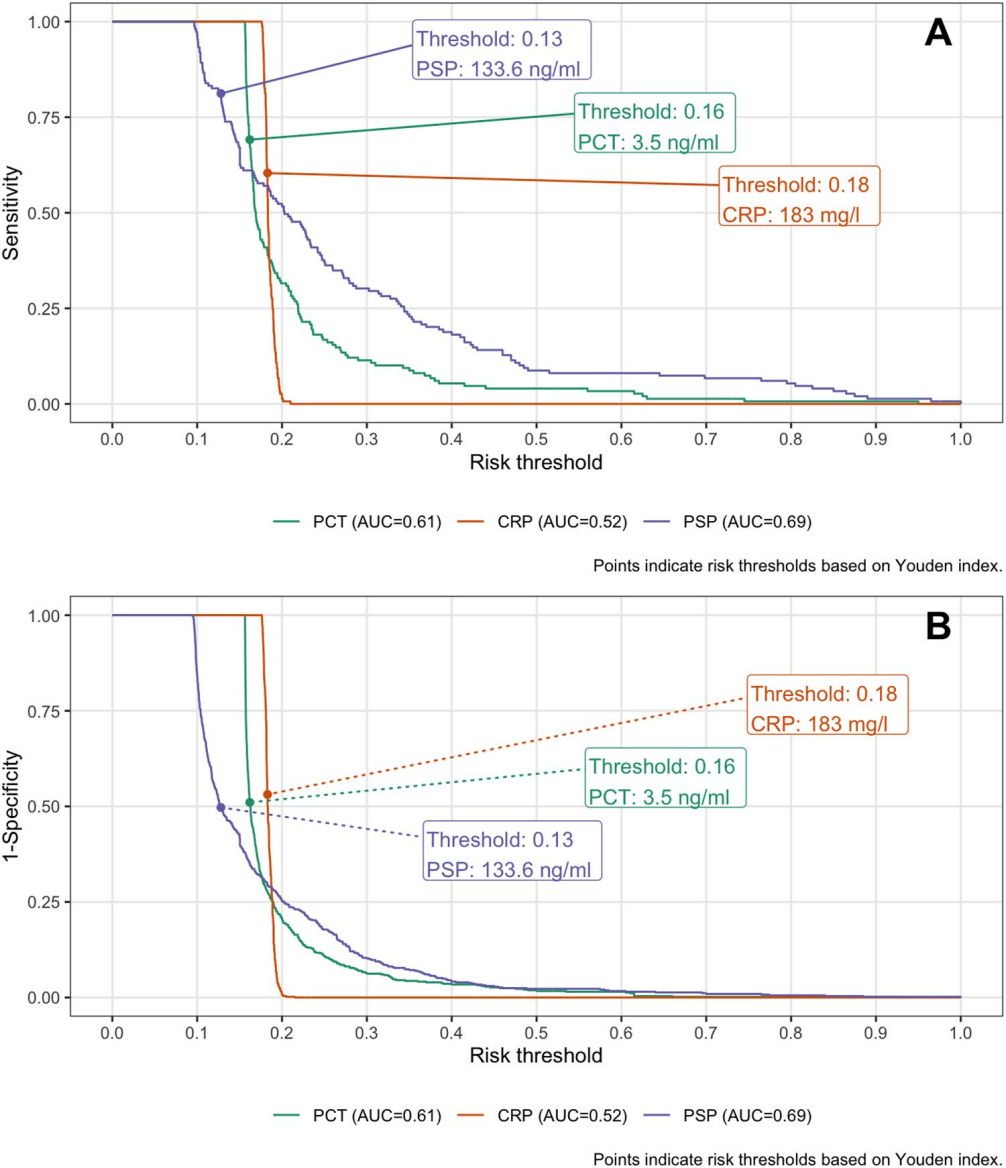
ICU mortality classification plot*.* Panel A: Sensitivity by risk threshold; Panel B: 1-Specificity by risk threshold; Risk threshold values shown for Youden index

PSP was strongly associated with ICU mortality (OR = 2.7, 95% CrI [1.3–6.0] per one SD increase), even after adjustment for age, gender and sepsis severity (OR = 1.5, 95% CrI [0.98–2.83], Supplemental Fig. 3). The AUC from an unadjusted RERS model was 0.69 [95%CI 0.64–0.74]. We identified a PSP cut-off value of 133.6 ng/ml based on Youden index at a risk threshold at 13% with positive (PPV, 0.32, 95%CI [0.27–0.36]) and negative (NPV, 0.90, 95%CI [0.87–0.93]) predictive values using PSP (Table 3). Calibration plots showed that the sensitivity for PSP was 0.96, 0.52, 0.30 for risk thresholds 10%, 20% and 30%; respective false positive rate values were 0.84, 0.25, 0.10 (Fig. 3). Similar analyses were performed for CRP and PCT. Combining biomarkers in all different models evaluated did not increase the discriminative performance of PSP (Supplemental Fig. 4; Supplemental Table 6).

**Table 3:** Discriminative measures at Youden’s index risk threshold for ICU mortality

Biomarker	AUC (95%CI)	PPV (95%CI)	NPV (95%CI)
PSP	0.69 (0.64, 0.74)	0.32 (0.27, 0.36)	0.9 (0.87, 0.93)
CRP	0.52 (0.47, 0.57)	0.24 (0.20, 0.29)	0.81 (0.76, 0.85)
PCT	0.61 (0.56, 0.66)	0.28 (0.23, 0.32)	0.85 (0.80, 0.88)

### Infection severity

**Fig. 4:**
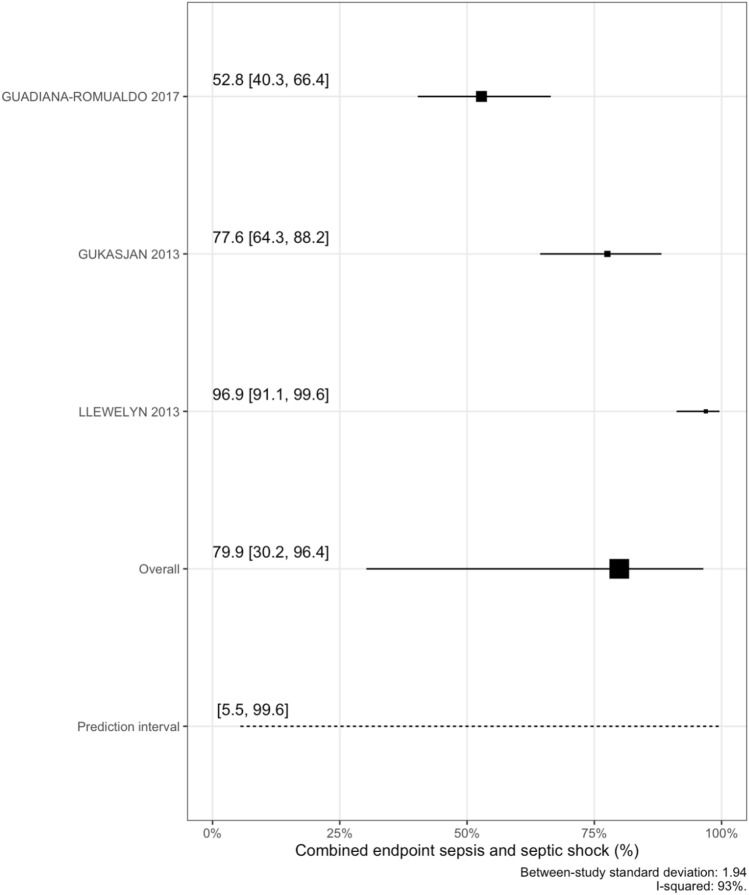
Combined endpoint sepsis and septic shock meta-analysis

PSP was higher in patients with sepsis/septic shock compared to those with mild infections and strongly associated with the combined endpoint of sepsis/septic shock in both unadjusted (OR = 11.4, 95% CrI [2.1–54.5]; per one SD increase and age–gender adjusted models (OR = 11.4, 95% CrI [1.9–48.9]), (Supplemental Fig. 5). For the secondary combined outcome of sepsis and septic shock we estimated a pooled overall percentage of 79.9%, with a 95% PI ranging from 5.5% to 99.6% with a considerable heterogeneity between studies (I-squared 93%), (Fig. 4).

**Fig. 5:**
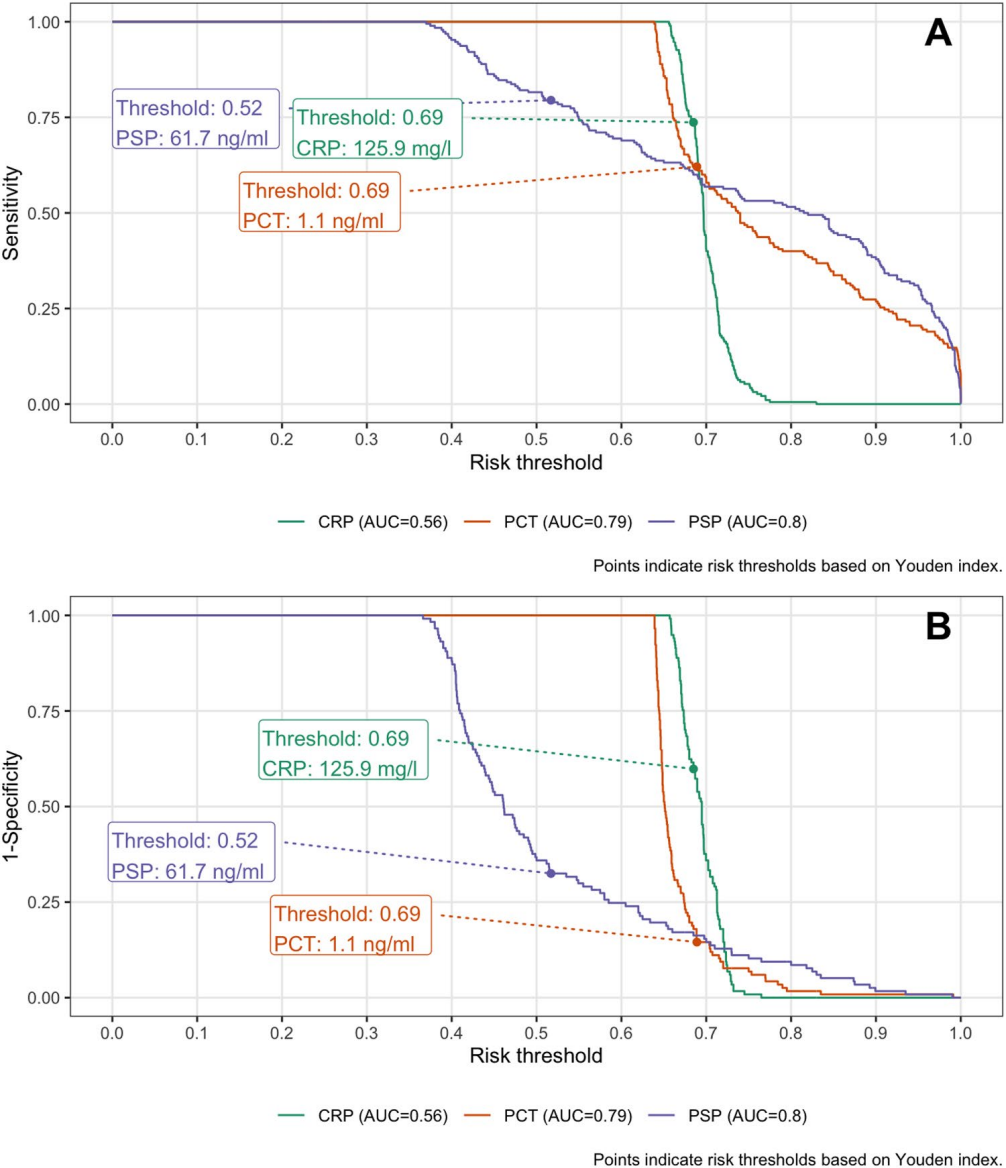
Combined endpoint sepsis and septic shock classification plot*. * Panel A: Sensitivity by risk threshold; Panel B: 1-Specificity by risk threshold; Risk threshold values shown for Youden index

Risk thresholds based on Youden index to discriminate mild infection form severe infection/septic shock were 61.7 ng/ml for PSP, 125.9 mg/l for CRP and 1.1 ng/ml for PCT (Fig. 5). Using those, PSP (AUC 0.80, 95%CI [0.75–0.85]) and PCT (AUC 0.79, 95%CI [0.74–0.84]) performed better that CRP (in stratifying patient according to infection severity: AUC was lowest for CRP (AUC 0.56, 95%CI [0.50–0.63]). PPV was the highest for PCT (0.87, 95%CI [0.81–0.92] and NPV for PSP (0.67, 95%CI [0.58–0.75]) (Supplemental Table 6). Discriminative performance (as measured by AUC) did not improve when biomarkers where combined (Supplemental Fig. 6; Supplemental Table 7).

## Discussion

We analyzed individual patient level data from five studies that measured PSP using the enzyme-linked immunosorbent assay technique investigating the diagnostic accuracy of PSP on ICU mortality and infection severity. Our results suggest that PSP has a very good discriminative ability, higher than CRP and comparable to PCT. To the best of our knowledge, the present study is the first meta-analysis of its kind using actual datasets from different studies on this very topic.

Correctly identifying patients suffering from severe sepsis or septic shock and predicting ICU mortality is key when treating patients with infection not only to rapidly stabilize the patient’s condition and positively influence outcome, but also to allocate an adequate amount of resources. It is also important for identification of appropriate patients for enrollment in trials of sepsis interventions. Current clinical scoring systems lack sensitivity and specificity to guide decisions and prognostication upon admission [36–38]. Despite their large use for comparing severity and predicting mortality across ICU patient populations, common ICU severity scores such as Acute Physiology and Chronic Health Evaluation (APACHE II) and Simplified Acute Physiology Score (SAPS II) are not designed to recognize and discriminate between individual outcomes [39]. Recently, the National Early Warning Score (NEWS) [40] has emerged as valuable tool to predict sepsis-related outcomes upon admission [41] or after ICU transfer [42]. Nowadays, NEWS has been incorporated almost universally in the UK in the patient management [43].

Besides their application to diagnose infection and assess the response to therapy, biomarkers are also increasingly being used to stratify patients according to their risk profiles and to predict sepsis-related outcomes [44]. For instance, certain blood transcriptomics of gene panels might accurately predict patient outcome after burn [45] or blunt trauma [46] and identify those at risk of developing infection in the course of recovery. On a larger scale, the performance of the widely available classical biomarkers CRP, PCT as predictors of adverse outcomes still remain controversial [47, 48].

The present study is the first individual patient level meta-analysis that systematically evaluates the performance of PSP in predicting infection severity and outcome in patients upon admission to ICU or ER. PSP demonstrated better predictive ability for ICU mortality in comparison to canonical biomarkers of infection as CRP, but similar to PCT. In addition, PSP could reliably stratify patients according to infection severity. Altogether, our data suggest that PSP could be used as a prognostic biomarker in such patients and support precision medicine in the management of infections and sepsis [49].

Better information on patients’ individual risk profile and outcome upon the admission to the ER or the ICU should assist healthcare givers and clinicians in their triage decision to make timely allocation of resources and therapeutic options. Correct identification of high-urgency patients avoids delays in the initiation of sepsis management, while reliable classification of low-urgency patients improve efficiency in the ER patient flow. Such approaches have been successfully evaluated in specific clinical settings such as urinary tract infections [50] as well as in the unselected patient populations (within the TRIAGE study) [51]. One advantage of PSP over other blood biomarker is the availability of a POC diagnostic tests using nanofluid technology, enabling rapid quantification of PSP at the bedside [17, 26, 52].

Our study has several strengths. First, we received individual patient level data from the eligible studies, which allowed us to model our study endpoints and biomarkers on patient level. Second, the original studies were performed in different centers across Europe and covered two clinical settings, including ER and ICU, which make the results more generalizable. Finally, the use of classification plots in contrast to conventional ROC allows for a direct visualization of the model’s discriminative ability enabling the clinicians to choose the threshold value according to the therapeutic question. A single threshold based on Youden index might be suboptimal from a clinical view, such that classification plots are a helpful tool to support clinicians in decision making. The main limitations of the meta-analysis are the relatively small numbers of included studies and the exclusion of newer ones performed using the recently available POC technology. Direct comparison with the previous ELISA technique with which all PSP levels were measured, is possible as POC PSP levels approximately equals 4.6 × previous ELISA ng/ml + 30 ng/ml [52].

## Conclusions

In conclusion, the present study confirms that PSP is a promising biomarker to predict sepsis-related outcome and estimate infection severity upon hospital and/or ICU admission. However, further prospective studies are needed to confirm its utility and safety in the daily clinical use.

### Supplementary Information

Below is the link to the electronic supplementary material.

Supplementary file1 (DOCX 876 KB)

## Data Availability

The Corresponding author has acces to all data included into the analysis. Requests should be submitted to the corresponding author in the first instance.

## Abbreviations

CI: Confidence interval
CrI: Credible interval
ER: Emergency room
CRP: C-reactive protein
ICU: Intensive care unit
PCT: Procalcitonin
PSP: Pancreatic stone protein
